# iCa^2+^ Flux, ROS and IL-10 Determines Cytotoxic, and Suppressor T Cell Functions in Chronic Human Viral Infections

**DOI:** 10.3389/fimmu.2020.00083

**Published:** 2020-03-06

**Authors:** Subhasmita Mohanty, Prakash Barik, Nagen Debata, Perumal Nagarajan, Satish Devadas

**Affiliations:** ^1^Infectious Disease Biology, Institute of Life Sciences, Bhubaneswar, India; ^2^Department of Pathology, Institute of Medical Sciences and SUM Hospital, Bhubaneswar, India; ^3^Experimental Animal Facility, National Institute of Immunology, New Delhi, India

**Keywords:** Cytotoxic T cell, Suppressor T cell, iCa2^+^ flux, IL-10, ROS

## Abstract

Exhaustion of CD8^+^ T cells and increased IL-10 production is well-known in chronic viral infections but mechanisms leading to loss of their cytotoxic capabilities and consequent exhaustion remain unclear. Exhausted CD8^+^T cells also called T suppressors are highly immune suppressive with altered T cell receptor signaling characteristics that mark it exclusively from their cytotoxic counterparts. Our study found that iCa^2+^ flux is reduced following T cell receptor activation in T suppressor cells when compared to their effector counterpart. Importantly chronic activation of murine cytotoxic CD8^+^ T cells lead to reduced iCa^2+^ influx, decreased IFN-γ and enhanced IL-10 production and this profile is mimicked in Tc1 cells upon reduction of iCa^2+^ flux by extracellular calcium channel inhibitors. Further reduced iCa^2+^ flux induced ROS which lead to IFN-γ reduction and increased IL-10 producing T suppressors through the STAT3—STAT5 axis. The above findings were substantiated by our human data where reduced iCa^2+^ flux in chronic Hepatitis infections displayed CD8^+^ T cells with low IFN-γ and increased IL-10 production. Importantly treatment with an antioxidant led to increased IFN-γ and reduced IL-10 production in human chronic Hep-B/C samples suggesting overall a proximal regulatory role for iCa^2+^ influx, ROS, and IL-10 in determining the effector/ suppressive axis of CD8^+^ T cells.

## Introduction

Cytotoxic T cell type1 (Tc1), a CD8^+^ T cell subset, plays crucial roles in clearing viral infections through the active secretion of cytolytic molecules and pro-inflammatory cytokines; specifically IFN-γ, TNF-α Perforin and Gr B (1–4). However, persistent antigenic presence as evidenced in chronic viral infections such as hepatitis B/C, HIV leads to T cell exhaustion characterized by sustained high expression (5–10) of surface inhibitory markers PD-1, Lag-3, CTLA4, and subsequent loss of cytotoxic capabilities (8–14). Elevated levels of IL-10 have also been reported in these infections (5, 6, 15–17) and multiple studies have reported a direct link between loss of cytotoxicity and increased levels of IL-10. The switching in cytokine production is also accompanied by altered expression of key Transcription Factors (18) such as T-bet, Eomesodermin (2, 19) indicating a complete phenotype change of Cytotoxic (Tc1) cells to a T suppressor (T sup) phenotype (17, 20). Inevitably domination of a suppressor phenotype has adverse pathological consequences not only limited to intra cellular infections but also to general immune homeostasis (10, 20–22) and subsequently this altered cytotoxic T cell balance completely breaks down immune response through exaggerated suppression. Partial restoration of cytotoxic function in exhausted CD8^+^ T cells has been reported by blockade of co-inhibitory receptors PD-1, Lag-3 as well as IL-10 receptors in *Lymphocytic choriomeningitis* and *Toxoplasma gondii* (5, 23) however the exact signaling pathway leading to conversion of effector CD8^+^ T cells into a T suppressor phenotype is yet undefined. Importantly elucidating the pathway of exhaustion will pave the way for targeting regulatory molecules that may help in complete restoration of function in suppressor T cells.

Different types of T sup cells execute their suppressor function through the following mechanisms: anti-inflammatory cytokine production, cell-cell contact mediated suppression and cytotoxicity to target cells and competitive consumption of IL-2 (24). For example CD8^+^CD28^−^ T sup cells execute their function by rendering APC tolerogenic, alloantigen-induced CD8^+^CD103^+^ T sup cells suppress T cell proliferation through cell to cell contact dependent mechanism and the CD8^+^CCR7^+^CD45RO^+^T sup cells function through IL-10. Also the naturally occurring T sup cells function through anti-inflammatory cytokine IL-10 (24, 25). Our study primarily focused on immune suppression through the anti-inflammatory cytokine IL-10 as our principal aim was to study the effect of chronic infection on TCR downstream signaling events, that eventually converted a pro-inflammatory cytokine producing effector CD8^+^ T cell into an anti-inflammatory cytokine producing T sup cells.

iCa^2+^ flux and ROS are two of the earliest signaling events downstream of TCR activation and while iCa^2+^ flux dynamics is reported to be decoded into differential cytokine production, the quantity of ROS is known to effect pro/anti-inflammatory cytokine production signaling pathways in CD4^+^ T cells (26–28). In T cells, the activation of T cell receptor (TCR) upon antigen presentation results in elevation of iCa^2+^ flux contributed by Ca^2**+**^ release from endoplasmic reticulum and Ca^2**+**^ influx through CRAC channels from extracellular source (23, 29). An increased window of iCa^2+^ is known to be required for NFAT1 translocation to the nucleus for transcription of IFN-γ (30, 31) and Gr B whose secretions are impaired in chronic infection(s) (17). Interestingly T Suppressor cells are known to induce functional suppression of CD8^+^ T cells through producing ROS in tumor microenvironment (32). Apart from this the co-inhibitory receptor PD-1 also leads to increase in cellular ROS that is reduced upon blockade of PD-1 (33). Importantly interplay between iCa^2+^ flux and ROS is known to positively or negatively regulate a number of signaling pathways (34, 35) depending upon the cell type, which has not yet been explored in chronic viral infection.

Considering the aforementioned facts we studied how iCa^2+^ flux and ROS interplay to convert pro-inflammatory response into an anti-inflammatory response. We observed that reduced iCa^2+^ flux leads to increased ROS production that in turn produced higher IL-10 and lower T-bet/IFN-γ in chronically activated CD8^+^ T cells through STAT3/STAT5 axis, whereas induction of ROS did not affect iCa^2+^ flux indicating a proximal regulatory role for iCa^2+^ flux. Further chronic Hep-B/C samples also displayed reduced iCa^2+^ flux and increased ROS as compared to their acute counterpart. Intriguingly treatment with a ROS scavenger was able to reduce IL-10 production and increase IFN-γ in CD8^+^ T cells from chronic Hep-B/C samples. Taken together our data suggest a proximal regulatory role for iCa^2+^ influx and ROS in determining the effector/ suppressive axis of CD8^+^ T cells.

## Materials and Methods

### Mice and Reagents

Wild type strain BALB/cJ, C57BL/6 ([N1]C57Bl/6J) and IL-10 KO (B6.129P2-Il10^tm1Cgn/J^) mice were obtained from Jackson Laboratories (Bar Harbor, ME), National Institute of Immunology (New Delhi, India) or from Imgenex (Bhubaneswar, India) and maintained in *12h-Dark-light cycle* in a pathogen free animal facility at Institute of Life Sciences, Bhubaneswar, with food and water provided *ad-libitum*. Age and sex matched inbred wild type BALB/cJ, C57BL/6, and or IL-10 KO mice were used for experiments and the ILS Institutional Animal Ethical Committee approved the present study.

Cytokine murine IL-12 was purchased from ProSpec (Rehovot, Israel) and recombinant human IL-2 from Imgenex Corporation, San Diego, CA, USA. All antibodies for activation and cytokine neutralization were procured from BioXcell, MA, USA. Fluorescent antibodies that include IFN-γ APC/Alexa fluor-488, IL-10 PE/Percp-Cy5.5/Alexa fluor-488, T-bet PerCP-Cy5.5, PD-1 PE-Cy7, Lag-3 PE, Granzyme-B –PE, Perforin-APC, Eomes-FITC, CD8-PerCP, CD69-PE-Cy7, CD62L-PE, pSTAT3-PE, pSTAT5-PEcy7 for intracellular and surface staining were purchased from e-Bioscience, San Diego, CA, USA and BD Biosciences, San Jose, CA, USA, Bio Legend, San Diego, CA, USA. NFAT1 antibody for confocal microscopy was purchased from Cell Signaling Technology (Danvers, MA, USA). Chemicals including Phorbol 12-myristate 13-acetate, Ionomycin, Brefeldin A, Bepridil Hydrochloride, Amlodipine Besylate, Thapsigargin, BTP2, and EDTA were obtained from Sigma Inc., USA.

### Human Peripheral Blood

The Institutional Human Ethical Committees of ILS, Bhubaneswar and SUM Hospital, Bhubaneswar, Odisha approved and sanctioned the present study. Briefly, blood samples from human healthy volunteers and acute (*n* = 14) /chronic Hep B (*n* = 4) patients were collected, where recruitment of healthy subjects was based on inclusion/exclusion criteria and Hep B subjects were based on confirmatory clinical diagnosis and tests. Acute and chronic Hep-B patients are confirmed by our clinical partner (chronic patients- HBsAg positive for >6 months, serum HBV DNA > 10^5^ copies/ml and persistent or intermittent increase in ALT/AST level). Samples were collected from Control, Hep B subjects with their informed consent and data of control and Hep B volunteers are depicted in a **Figure 2D**. Viral Load (signal cut off ratio), Alkaline Phosphatase, Glutamic-oxalacetic transaminase (SGOT) and glutamic-pyruvic transaminase (SGPT) were also assayed to confirm and co relate infection status and extent of liver damage.

### Cell Isolation and *in vitro* Differentiation of Cytotoxic T Cells

CD8^+^ T cells were isolated from spleens and lymph nodes of 8–12 weeks old C57/BL6 mice by negative selection using Dynal beads (Invitrogen, MA, USA) according to the manufacturer's protocol. Cell purity was checked by flow cytometry and was consistently around ~90%. Isolated cells were cultured in RPMI 1640 supplemented with 10% fetal bovine serum, Australian origin (PAN-Biotech), 100 U/ml penicillin, 100 ug/ml streptomycin and 50 mM 2-mercaptoethanol (Sigma). For Tc1 differentiation, CD8^+^ T cells at 1 x 10^6^ concentration was activated with plate bound anti CD3 (1 ug/ml) and anti CD28 (2 ug/ml) and provided with IL-12 (10 ng/mL), anti-IL-4 (10 mg/mL), and 100 IU/ml of IL-2 was added after 24 h. After 3 days cells were removed from activation signals and were rested for 2 days with 100 IU/ml h IL-2. For chronic activation the differentiated Tc1 cells were re plated at 1 × 10^6^ cells/ml every third day on plate bound anti CD3 (1 ug/ml) and anti CD28 (2 ug/ml) for 13–22 days and supplemented with 100 IU/ml. After 13 days cells were removed from activation signals and were rested for 2 days with 100 IU/ml h IL-2. Cells were then washed with RPMI 1640 media and used for subsequent experiments as indicated. Cytotoxic T cell (Tc) differentiation state was confirmed by detecting signature transcription factor and cytokines by Flow Cytometry.

### Flow Cytometry

Differentiated cytotoxic T cells at 1 × 10^6^/ml were reactivated with 50 ng/ml PMA and 1 ug/ml Ionomycin for 8 h for detecting intracellular cytokines, transcription factors and surface markers. 10 ug/ml Brefeldin- A was added to the culture for the last 4 h and dead cells were excluded using Live Dead Fixable violet Dead Cell stain kit (Invitrogen, MA, USA). Intracellular cytokine staining was performed using Cytofix/Cytoperm Fixation/Permeabilization Solution Kit (BD Biosciences, San Jose, CA, USA), while transcription factor staining was performed using FOXP3 staining buffer set (eBioscience, San Diego, CA, USA) according to the manufacturer's instructions. For inhibitor studies, cells were incubated with the mentioned inhibitors for 15 min prior to stimulation with PMA/Ion.

The antibodies used were anti-IFN-γ-APC or Alexa flour 488, anti-IL-10-PerCP/Cy5.5 or Alexa Fluor 488, anti-T-bet-PerCP/CY5.5 or eFluor 660, anti-FOXP3- Alexa Fluor 647, PD-1-PE Cy7, Lag3-PE, anti ERK (Thr202/Tyr204), anti p38 (Thr180/Tyr182) PE, antiCD45RO PE-CF594, and antiCD45RA APC-Cy7. All antibodies including their corresponding isotypes were purchased from eBioscience (San Diego, CA, USA) or BD Pharmingen (San Jose, CA). Data was acquired on a BD-LSR II (BD Biosciences, San Jose, CA, USA) and analyzed with FACS Diva Software. For intracellular staining of STAT-3 and STAT-5, CD8^+^ T cells were stimulated with PMA/Ion for 3 h. and were fixed with formaldehyde for 30 min at room temperature. The cells were permeabilized with ice-cold methanol with vigorous vortexing followed by incubation at 4°C for 10 min. Cells are then washed and stained with antibodies for 1 h at 4°C.

### Confocal Microscopy

Cytotoxic and or suppressor T cells were differentiated and confirmed according to the protocol described above. Following activation of these cells for 1 h, they were washed with PBS and fixed for 20 min. Cells were then washed to remove fixation buffer and was followed by Permeabilization and were then stained with primary antibody anti NFAT1 on ice, followed by secondary antibody (Alexa flour 555). The unbound and extra antibodies were washed off and nuclei were stained with DAPI in the dark for about 20 min. Thereafter NFAT1 translocation to nucleus was checked in TCS SP5 Leica confocal microscope using the 488 and 355 nm lasers.

### Calcium Kinetics Assay

10 × 10^6^ Tc1 cells were stained with 3 uM Fluo-4 AM and incubated for 45 min at room temperature followed by washing with DPBS (w/o Ca^2+^ and Mg^2+^) to remove unbound and excess stain and were finally re suspended in DPBS (with Ca^2+^ and Mg^2+^). Base line value for iCa^2+^ flux was acquired for 1 min and then cells were treated with 5 um Bepridil hydrochloride or 100 nm BTP2 or 25 nm Amlodipine or 0.5 mM EDTA for 15 min followed by TCR stimulation with PMA/Ion. Ca^2+^ kinetics was acquired for a total of 30 min in a BD-LSRII (BD Biosciences) at 540 nm and analyzed with Flow-Jo software. We also did the experiment through secondary cross-linking by anti CD3 and found a similar trend as that of PMA/Ion.

### Cytotoxic Protein Assay

For identifying the expression of cytotoxic molecules that include Granzyme-B and Perforin, differentiated T cytotoxic cells at 1 x 10^6^/ml were reactivated with 50 ng/ml PMA and 1 ug/ml Ionomycin for 7 h. 10 ug/ml Brefeldin-A was added to the culture during the last 4 h. Dead cells were excluded using Live Dead Fixable violet Dead Cell stain kit, with 405 nm excitation (Invitrogen, MA, USA). Intracellular cytokine staining was performed using Cytofix/Cytoperm Fixation/Permeabilization Solution Kit (36) as per the manufacturer's protocol. The antibodies used were anti-Granzyme B-PE and anti-Perforin APC. Data was acquired on a BD LSR II (BD Biosciences) and analyzed with FACS Diva Software.

### Detection of Intracellular Reactive Oxygen Species (ROS)

Intracellular ROS levels were measured using oxidation sensitive fluorescent dyes, Dihydroethidium (DHE). For detecting intracellular superoxide, differentiated Tc1 and Tc15 cells were pre-loaded with DHE (5 uM) for 30 min in medium without serum. Cells were then washed with PBS and treated with indicated concentrations of Menadione for 15 min with or without NAC (5 mM). In some cases, cells were treated with Menadione at indicated concentration (2.5 uM) for 15 min and then DHE (5 uM) was added to the culture and incubated further for 15 min. After incubation, cells were washed, resuspended in PBS and oxidized DHE was detected using Flow cytometer.

### Intracellular Phospho-Protein Staining

For intracellular staining of human phospho-STAT3/5/6, unstimulated or stimulated CD8^+^ T cells were fixed with formaldehyde (final concentration of 1.5%) for 30 min at room temperature. They were then permeabilized with ice-cold methanol with vigorous vortexing and incubated at 4°C for 24 h. After washing with staining media (PBS with 1% BSA), cells were stained with respective antibodies for 1 h at room temperature and samples acquired in Flow cytometer.

### Cytotoxic Assay

For assessing the functionality of chronically activated CD8^+^ T cells (Tc15), Tc1 and Tc15 cells were taken at 1 million/ml concentration and co-cultured with CD4^+^ T cells (target cells) at 2:1 (CD8^+^: CD4^+^) ratio and stimulated with PMA (50 ng/ml)/Ion (1 ug/ml) for 8 h. The CD4^+^ T cells were labeled with 1 um CFSE. After incubation the cells were stained with CD4, CD8, and Live Dead Fixable violet Dead Cell stain kit, with 405 nm excitation (Invitrogen, MA, USA). The CD4^+^ T cells were analyzed for loss of CFSE and DCD+ or both in presence or absence of PMA/Ion treatment and with/without CD8+ T cells (Tc1 and Tc15 as Effector cells).

### IL-10 Rescue Experiment

Tc15 cells were taken at 1 million/ml concentration and stimulated with PMA/Ion as previously described. Recombinant mouse IL-10 (proSpec, Israel) is added exogenously at 200 ng/ml and 400 ng/ml concentration and incubated for 8 h. BFA was added at 10 ug/ml concentration after 6 h of incubation, cells were stained with anti-IFN-γ antibody, acquired and analyzed on BD LSR Fortessa.

### Statistics

Statistical analysis was performed using two-way ANOVA followed by Bonferroni's multiple comparison tests when comparing multiple groups. Mann-Whitney U test was performed for human samples using Prism 5.0 (Graph Pad, La Jolla, CA, USA).

## Results

### Reciprocal IFN-γ and IL-10 Production in Acute and Chronic Human Hep-B Patients

**Figure 1:**
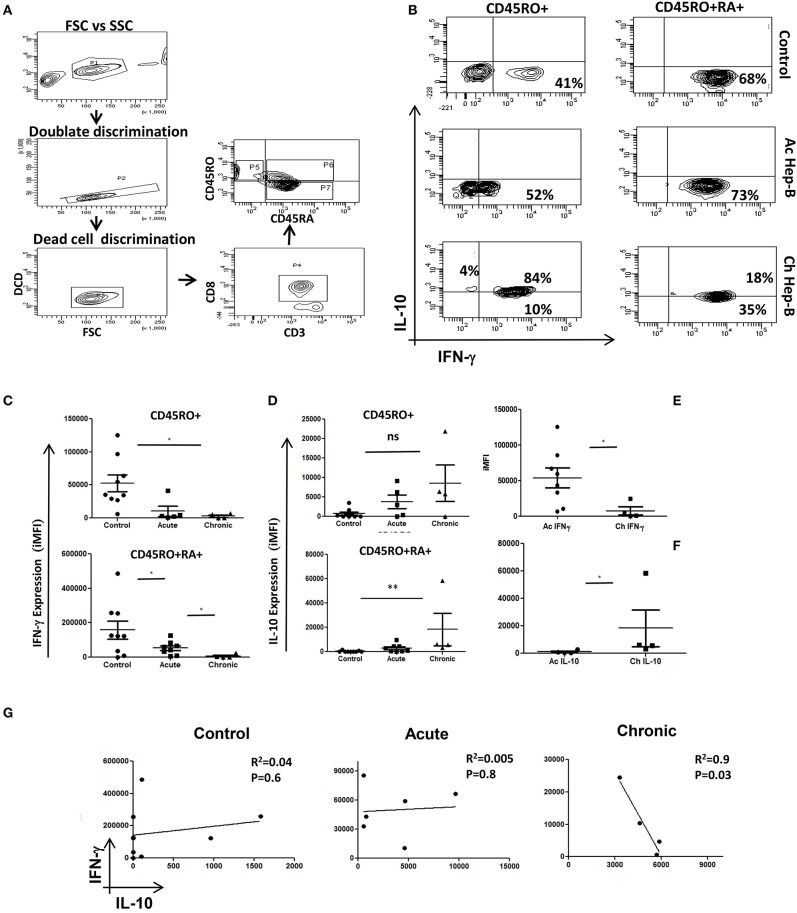
Acute and Chronic Hep B patients' display inverse IFN-γ and IL-10 secretion patterns: CD8^+^ T cells were isolated from PBMC of control, acute or chronic Hep B/C patients and stimulated with PMA/Ion for 6 h and stained for cytokines as described elsewhere. A schematic of gating strategy is represented in **(A)**. **(B)** Representative figure showing IFN-γ vs. IL-10 production in CD45 RO^+^/RO^+^RA^+^ phenotypes in control, acute Hep B/C and chronic Hep B/C patients. **(C)** A summary of IFN-γ and IL-10 production by CD8^+^ T cells from acute (*n* = 8) or chronic Hep-B/C patients (*n* = 4). **(D)** A summary of IFN-γ and IL-10 production by effector (CD45RO^+^ CD45RA^+^) and memory (CD45RO^+^) subsets from control (*n* = 9), acute (*n* = 8) or chronic Hep-B/C patients (*n* = 4). **(E,F)** Comparison of IFN-γ and IL-10 production by CD8^+^ T cells of acute and chronic Hep-B patients. Bar represents iMFI for respective cytokines. All samples were tested for significance by Mann-Whitney test and *p* < 0.5 (*p < 0.05,**p < 0.01, ***p < 0.001) considered as significant. Regression analysis of IL-10 vs. IFN-γ in control, acute and chronic Hep B is shown **(G)**.

We examined and confirmed for loss of cytotoxic function and gain of immune-suppression in Memory and Effector CD8^+^ T cells from chronic Hep-B patients (Figure 1B) by gating the cells as described (Figure 1A) to primarily establish that chronic infections/activation severely impairs cytotoxic T cell functions whilst converting them to a suppressor phenotype. CD8^+^ T cells isolated from acute Hep-B patients actively secreted IFN-γ (*P* = 0.0162) and were clearly contrasted from CD8^+^ T cells derived from chronic Hep-B patients (*n* = 4) that secreted higher IL-10 (*P* = 0.0286) (Figures 1B,E,F). Cytokine analyses between control and acute, effector and memory compartments clearly demonstrated that IFN-γ was significantly higher (*P* = 0.0253) in both acute compartments (Figure 1C). Not surprisingly IFN-γ production from effector and memory compartments of the two were significantly higher (*P* = 0.0253) than their chronic counterpart. Whereas, though anti-inflammatory cytokine IL-10 production was significantly higher (*P* = 0.0037) in effector compartment of CD8^+^ T cells isolated from chronic Hep-B patients, the difference was insignificant (Figure 1D) in memory compartment and suggested that the cytotoxic effector function of the effector compartment was more affected. In direct contrast Control subjects displayed a strong cytotoxic phenotype with similar IFN-γ production to acute subjects (Figures 1B,C) suggesting an early and appropriate cytotoxic response phase in both. Regression analysis determination to analyze for relationship between IL-10 and IFN-γ production displayed an inverse relationship in chronic Hep B (Figure 1G). However the regression analysis in control and acute Hep-B patients show very weak relationship between the two cytokines. Thus, our data suggested that cytotoxic T cell capabilities are or maybe progressively lost during chronic Hep-B infection with a concomitant increase in IL-10 production that in turn could further reduce pro inflammatory status contributing to persistence of infection. Undetectable pathogen and or basal presence in “control” humans do not aid this conversion nor block activation of CD8^+^ T cells as seen in both controls and acute Hep B patients. While comparable IFN-γ production was noticed in the effector compartment in most acute patients, crucially it was not suggestive of chronicity or imminent immune suppression. Thus, it is implicit from these results that simple reduction of IFN-γ and increased production of IL-10 during viral infection(s) alone do not lead to suppressed immune system or to disease chronicity.

### Impaired iCa^2+^ Influx in Chronic Hep B Patients

**Figure 2:**
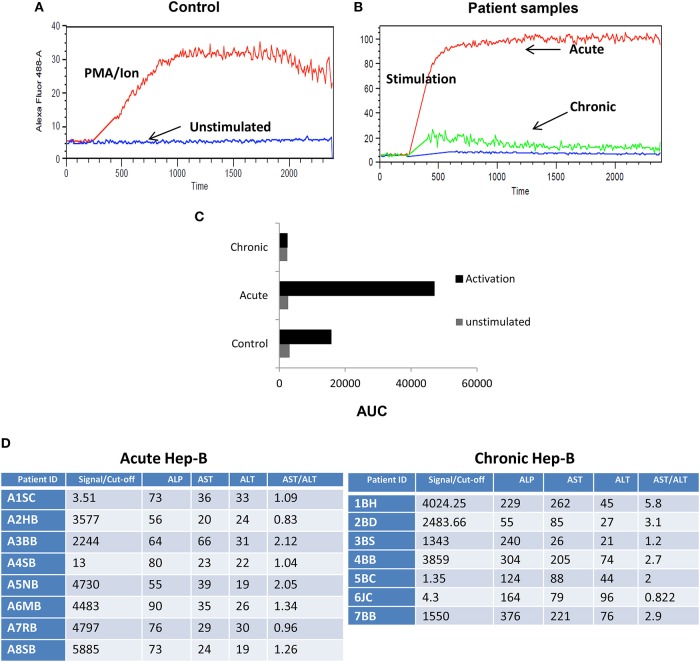
Impaired iCa^2+^ influx in chronic Hep B/C patients. CD8^+^ T cells from control were isolated and stimulated in the presence or absence of CD3 or PMA/Ionomycin to examine for basal and stimulated iCa^2+^ influx **(A)** Unstimulated (US) cells from controls did not display any iCa^2+^ influx as compared to CD3 or PMA/Ionomycin stimulation. CD8^+^ T cells were isolated from Control, acute or chronic Hep B/Hep C patients followed by staining with Fluo-4 AM and iCa^2+^ influx was recorded for 10 min upon PMA/Ion stimulation. Figure showing iCa^2+^ influx in control, acute and chronic Hep-B/C patients **(B)**. **(C)** Cumulative data for area under curve of iCa^2+^ influx in control, acute and chronic Hep-B/C patients from three different experiments. **(D)** Table shown is for confirmation of acute and chronic viral hepatitis as assayed by viral load and Liver function tests.

Post-acute and chronic infection confirmation, we examined for early TCR downstream events that dictate the switch from a cytotoxic response to an immune suppressive one. We compared iCa^2+^ influx (Figures 2A–C) an early TCR downstream signaling event following CD8^+^ T cell activation in acute and chronic Hepatitis B (Hep B) and Hepatitis C (Hep C) (data not shown) patients from their CD8^+^ T cells. As base and for “controls” we first examined negatively selected CD8^+^ T cells from healthy controls for their iCa^2+^ influx (Figure 2A) when challenged with or without PMA/Ion and TCR activation by secondary crosslinking and not surprisingly CD8^+^ T cells displayed a robust iCa^2+^ influx (Figure 2A) which co related well with cytokine response too (Figures 1A,B). We then compared Ca^2+^ kinetics following PMA/Ion in chronic Hep B or Hep C patients with their acute counterparts, which showed that CD8^+^ T cell iCa^2+^ influx was completely abrogated in chronic patients, and resembled “unstimulated” controls (Figures 2A,B). The increased switch to an immuno-suppressive profile was also co related well with their clinical diagnostic enzyme profile including Alkaline phosphatase (37), alanine transaminase (38) and aspartate transaminase (AST) (Figure 2D). The reduced iCa^2+^ influx in chronic Hep B/ Hep C derived CD8^+^ T cells suggested a significant change in early TCR down-stream events, specifically iCa^2+^ influx, following persistent viral infection that effects pro/suppressive cytokine secretion such as IFN-γ and IL-10. Our data including data from other labs indicated that duration, amplitude, frequency and concentration of iCa^2+^ flux (39) could lead to differential signaling outcomes.

### Chronic TCR Activation of Tc1 Cells Leads to Impaired Cytotoxic Function and Increased IL-10 Production

**Figure 3:**
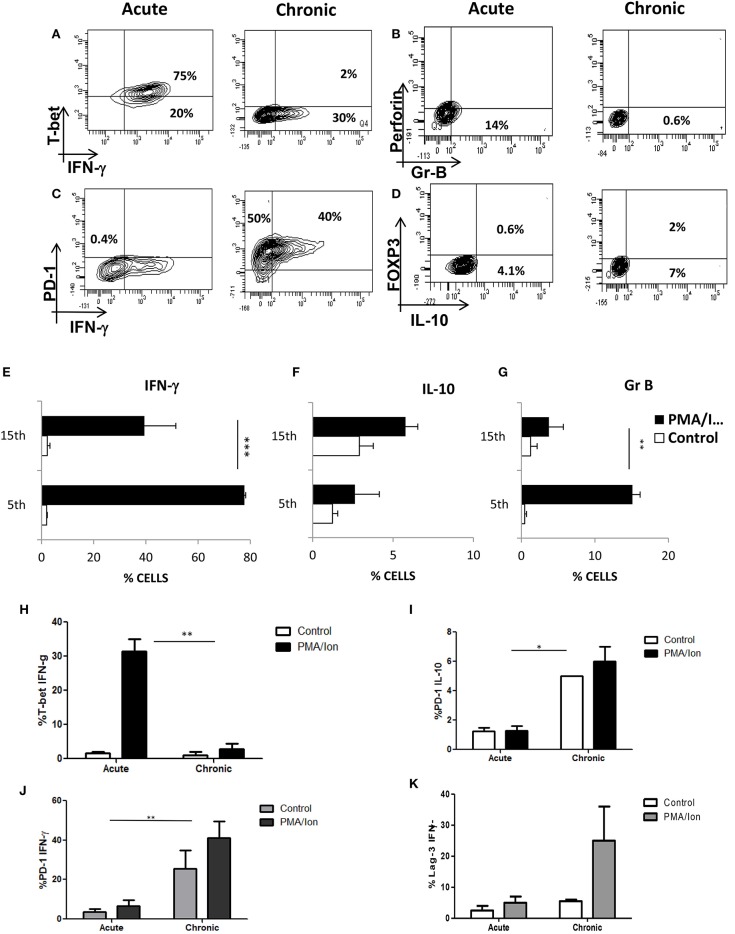
Chronic activation of Tc1 type leads to impaired cytotoxic function and increased IL-10 production. Murine CD8^+^ T cells were negatively selected and activated with CD3/28 for 5 days (Acute) or 15 days (Chronic). These cells were then re activated with PMA/Ionomycin for about 8 h and analyzed for multiple proteins. Representative figures for IFN-γ vs. T-bet **(A,E,H)**, Gr B vs. Perforin **(B,G)**, IFN-γ vs. PD-1 **(C,J)** and IL-10 vs. FOXP3 **(D,F)** and cumulative data for the frequency of Tc1 and Tc15 cells producing IFN-γ **(I)**, IL-10 **(J)** and Gr B **(K)** are shown. Loss and gain of cytotoxic and Immuno suppressive functions were analyzed and confirmed during acute and chronic activation. Bars representing % T-bet^+^IFN-γ^+^ **(H)**, PD-1^+^IL-10^+^ **(I)**, PD-1^+^ IFN-γ^+^ **(J)** Lag-3^+^IFN-γ^+^ **(K)** cells are shown for acute and chronically activated Tc1 cells. All samples were tested for significance by using two-way ANOVA and *p* < 0.5 (*p < 0.05, **p < 0.01, ***p < 0.001) considered as significant.

**Figure 4:**
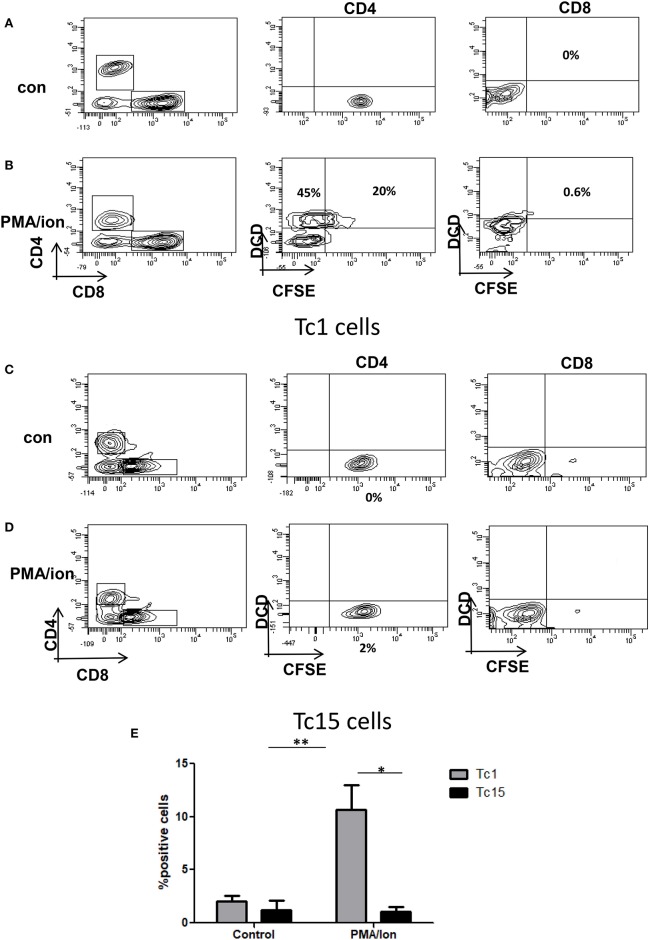
Impaired cytotoxic function in Tc15 cells. The functionality of Tc1 and Tc15 cells were compared by co-culturing them with CFSE stained CD4+ T cells at 2:1 ratio and stimulated with PMA/Ion for 8 h. After incubation the cells were stained for dead cell discriminator dye. Representative figures showing **(A)** No death and CFSE loss in unstimulated control of Tc1 cells. **(B)** With PMA/Ion stimulation the CFSE+ DCD+ CD4 T cells were 45% and DCD+ CD4+T cells were 20%. **(C,D)** Co-culture of CD4+ T cells with Tc15 cells show no death in control and 2% DCD+CFSE+ with PMA/Ion stimulation. **(E)** Derived data showing significantly lower death in CD4+ T cells when co-cultured with Tc15 cells as compared to their Tc1 counterpart. All samples were tested for significance by using two-way ANOVA and *p* < 0.5 (*p < 0.05, **p < 0.01, ***p < 0.001) considered as significant.

To further dissect how TCR activated iCa^2+^ influx could regulate IFN-γ/IL-10 production, we established cytotoxic and T suppressor phenotypes (Figures 3A–H) from negatively selected murine CD8^+^ T cells. Signature cytokine(s), Transcription factor(s), cytolytic protein(s) and surface co-receptor(s) including IFN-γ,TNF-α, T-bet, Eomesodermin and FoxP3, Gr B, and Perforin, PD-1, Lag-3 were examined to establish and monitor classically or acutely activated (day 5) Cytotoxic T cells and chronically activated (day 15) T suppressor phenotypes. The above mentioned multi-parameters were able to establish the said phenotypes when we examined cytotoxic T cells at defined intervals and amongst chronic activation profiles, Day 15 was chosen, as the parameters studied showed very significant loss of cytotoxicity and gain of immune suppression. We report that IFN-γ (*P* = 0.0051), Gr B (P = 0.03) and Perforin expression were significantly reduced in chronic activation as compared to its acute counterpart (Figures 3A,B,E,G), also their ability to kill the target cells (CD4^+^ T cells) was significantly reduced as compared to their acute counterpart (Figures 4A–E) further confirming chronic activation induced loss of functionality in Tc15 cells. Concomitantly the expression of surface co-inhibitory receptors PD-1 (*P* = 0.0126) and Lag-3 were significantly increased thus confirming loss of cytotoxic capabilities and gain of immune suppression (Figures 3D,K). The increased expression of surface inhibitory receptors, loss of inflammatory cytokines, cytotoxic proteins and related transcription factor in exhausted T cells during chronic activation indicated gradual CD8^+^ T cell exhaustion due to persistent *ex vivo* activation. The reduction of pro-inflammatory cytokine IFN-γ correlated well with the reduced expression of its master transcription factor T-bet (Figures 3A,H) and this process was accompanied by an increased secretion of anti-inflammatory cytokine IL-10 (Figures 3D,F). Further analysis also suggested that even though T-bet^+^ IFN-γ^+^ (Figure 3H) and IFN-γ^+^ population were significantly reduced in chronic activation, PD-1^+^ IFN-γ^+^ (Figure 3J) population had also increased significantly as compared to their acute counterpart and this could be clearly attributed to significantly higher expression of PD-1. We also found that PD-1^+^ IL-10^+^ (Figure 3I) had significantly increased as compared to their acute counterpart and suggested that all IL-10 producing cells express the co-inhibitory receptor PD-1, marker of T cell exhaustion, during chronic activation. These results suggest that chronic activation of CD8^+^ T cells may alter downstream TCR signaling events that eventually lead to a phenotype change from Tc1 subtype to a T suppressor type and a flip of pro-inflammatory cytokine IFN-γ to anti-inflammatory cytokine IL-10. Most significantly these parameter changes allowed us to monitor and confirm loss of cytotoxicity and gain of immune suppressive functions in CD8^+^ T cells.

### Reduced iCa^2+^ Influx Leads to Decreased NFAT1 Translocation to Nucleus in Tc15 Cells

**Figure 5:**
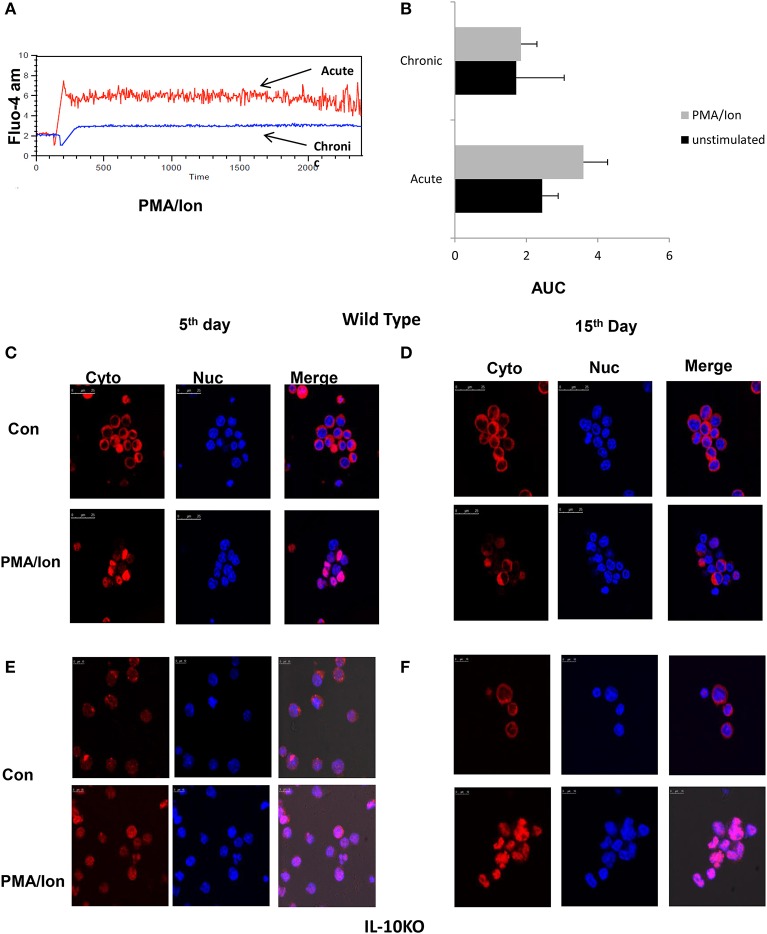
Reduced iCa^2+^ influx and inhibition lead to NFAT1 translocation to nucleus in Tc15 cells. (i). Murine Tc1 and Tc15 cells differentiated and characterized as described elsewhere were labeled with Fluo-4 AM for 45 min and washed with DPBS and re suspended in DPBS (w/o-Ca^2+^, Mg^2+^). A 1 min basal reading was taken followed by stimulation with PMA/Ion and then iCa^2+^ kinetics were checked for about 30 min **(A)**. Unstimulated basal (not shown) and PMA/Ion stimulated Acute (Tc1) and Chronic (Tc15) are shown. Bars representing iCa^2+^ influx of Tc1 and Tc15 cells' Area Under Curve from three different experiments are shown **(B)**. For translocation studies Tc1 **(C,E)** and Tc15 **(D,F)** from Wild type and IL-10 KO mice were incubated with PMA/Ion for 1 h and stained with NFAT1 antibody and DAPI. The cells were then analyzed on a Leica confocal Microscope.

To delineate and differentiate the signaling cascade following TCR activation that leads to switching off of IFN-γ and to secretion of IL-10, we first compared iCa^2+^ influx, one of the most proximal signaling events following TCR activation. Our hypothesis was based on earlier studies that establish the dynamics of iCa^2+^ flux as a deciding factor for the signaling outcome (40, 41). We observed that iCa^2+^ influx was significantly higher in acute Tc1 as compared to their chronic counterpart (Figures 5A,B). Further, iCa^2+^ concentration for a given period of time as assayed by their Area Under Curve (42), depicted a decrease in iCa^2+^ concentration in chronically activated Tc1 cells as compared to their acute counterparts (Figures 5A,B). Significantly stimulated increase between “unstimulated” control and chronically stimulated CD8^+^ T cells were almost negligible, strongly suggesting a complete insensitivity or near shut down of iCa^2+^ influx. Our results suggested that iCa^2+^ influx downstream TCR signaling was altered during chronic activation and could significantly impact cytotoxic functions. Consequently we also checked for NFAT1, a widely reported Ca^2+^ dependent transcription factor for IFN-γ secretion in Tc1 cells (28, 31, 43) by nuclear translocation assays to determine the effect of a reduced window of iCa^2+^. Not surprisingly we found that NFAT1 translocation to the nucleus was significantly reduced during chronic activation of Tc1 cells (Figures 5C,D) and thus we inferred from these results and from reduced T-bet expression (Figures 3E,L) that reduced iCa^2+^ influx could alter IFN-γ production at the transcriptional level. We then performed NFAT1 translocation studies from chronically activated CD8^+^ T cells derived from IL-10 KO mice (Figures 5E,F) and found significant translocation comparable to wild type. The IL-10 KO translocation study clearly proved that distal IL-10 was responsible for negatively regulating NFAT1 translocation and the subsequent IFN-γ production. Taken together these results show an intricate iCa^2+^-IL10 axis that finely down regulates NFAT1 translocation to the nucleus and subsequent IFN-γ production, and a possible involvement in the conversion of the cytotoxic T cell to a suppressor phenotype.

### Reduction of iCa^2+^ Leads to Decrease IFN-γ and Increased IL-10 Production During Acute Activation

**Figure 6:**
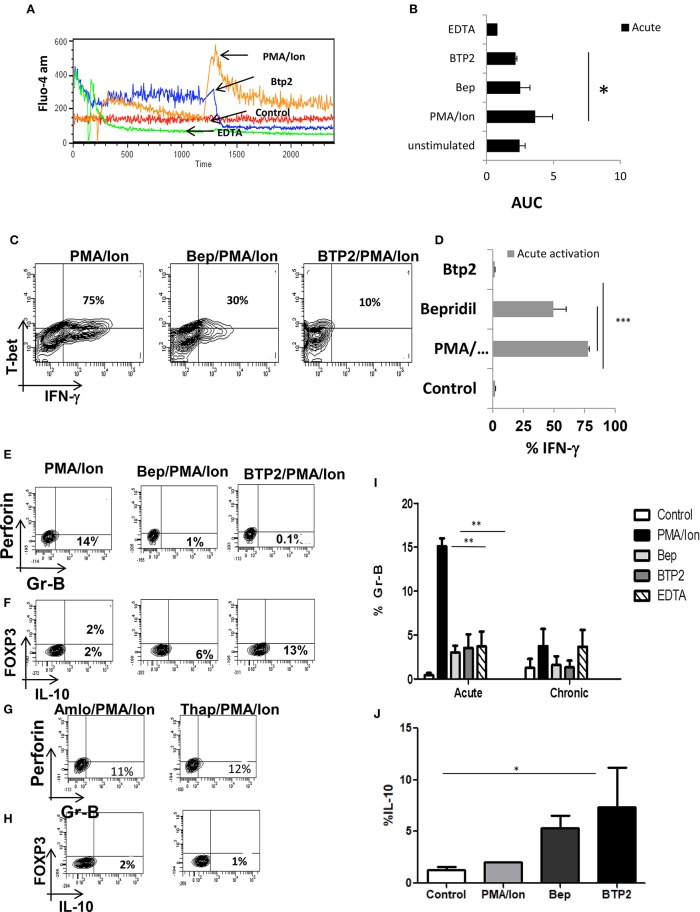
Reduction of iCa^2+^ leads to decrease IFN-γ production in Tc1 cells. (i) Differentiated Tc1 cells were treated with the mentioned inhibitors for 15 min and then stained with Fluo-4- AM for 45 min. Representative figure is shown for comparative iCa^2+^ influx of PMA/Ion vs. BTP2 vs. EDTA vs. unstimulated **(A)** and cumulative data for area under curve from 3 different experiments are depicted **(B)**. Differentiated Tc1 cells were treated with the mentioned inhibitors for 15 min followed by reactivation with PMA/Ion for 8 h and analyzed for cytotoxic T cell markers. Representative figures for IFN-γ vs. T-bet production in PMA/Ion vs. Bep/PMA/Ion vs. BTP2/PMA/Ion **(C)** are shown. Cumulative data from 3 different experiments for frequency of Tc1 cells producing IFN-γ is shown **(D)**. Differentiated Tc1 cells were treated with the mentioned inhibitors for 15 min followed by reactivation with PMA/Ion for 8 h. Representative figures for Gr B vs. Perforin and IL-10 vs. FOXP3 production in PMA/Ion vs. Bep/PMA/Ion vs. BTP2/PMA/Ion **(E,F)** and Amlo/PMA/Ion vs. Thap/PMA/Ion **(G,H)** are shown. Summary of the frequency of Tc1 and Tc15 cells producing Gr B **(I)** and IL-10 **(J)** are shown. All samples were tested for significance by using two-way ANOVA and *p* < 0.5 (*p < 0.05, **p < 0.01, ***p < 0.001) considered as significant.

Chronic activation of cytotoxic T cells lead to loss of cytotoxic function and gain of immune suppressive capabilities and was marked by altered iCa^2+^ influx, significant decrease in IFN-γ and Gr B and increase in IL-10. The role of IL-10, PD-1, Lag-3, and NFAT-1 gave further proof and strengthened our hypothesis for an iCa^2+^-NFAT1-IL-10 axis in balancing cytotoxicity and immune suppression. To further define and differentiate the signaling cascade and ascertain the regulatory role of iCa^2+^ dynamics following TCR activation that leads to switching off of pro-inflammatory cytokine IFN-γ and secretion of anti-inflammatory cytokine IL-10, we altered iCa^2+^ influx through specific inhibitors to examine for iCa^2+^'s role as one of the most proximal TCR signaling events. Amongst several inhibitors used, Bepridil hydrochloride, (Bep) a non-selective plasma membrane calcium channel inhibitor and BTP2, a CRAC channel inhibitor had the most significant inhibitory effects. Reduction of iCa^2+^ influx (Figures 6A,B) though Bepridil hydrochloride and BTP2 reduced IFN-γ and Gr B production (Figures 6B–G) while reduction with Amlo and Tg, was not significant. This suggested that iCa^2+^ flux through extracellular calcium channel played a significant role on pro-inflammatory cytokine IFN-γ production. We also found that whereas the pro-inflammatory cytokine IFN-γ was significantly reduced, the anti-inflammatory cytokine IL-10 was increased proving that modulating iCa^2+^ flux did not simply shut down cellular processes. IL-10 production was increased with Bep and BTP2 where the increase recorded was 6 and 13%, respectively and suggested a finely tuned iCa^2+^ flux regulating IFN-γ and IL-10 production upon inhibition of specific calcium channels. We also found that extracellular calcium channel inhibition with Bep and BTP2 led to significantly reduced Gr-B expression (Figure 6E) whereas inhibition of intracellular calcium channels through Amlo and Tg had no effect on Gr-B expression (Figure 6G). This suggested that Gr B reduction was inversely co related with IL-10 production as when IL-10 production was higher with Bep and BTP2, Gr-B expression reduced significantly but their expression was unaffected in absence of significantly higher IL-10 through intracellular calcium channel inhibition. These results together suggest that inhibition of iCa^2+^ influx during acute activation could mimic chronic activation profiles with respect to IFN-γ, Gr B and IL-10 production while also strongly suggesting that IL-10 negatively regulated Gr B expression in Tc1 cells. The significant loss of cytotoxic proteins Gr B and perforin confirmed the seminal and direct role for iCa^2+^ influx in establishing and propagating cytotoxic function while the switch to IL-10 production by use of the iCa^2+^ influx inhibitors complemented and completed its seminal role. Most significantly the results from iCa^2+^ influx inhibitors and the iCa^2+^ influx profile from chronically activated CD8^+^ T cells clearly suggest an exquisite co-relation between iCa^2+^ influx and pro and anti-inflammatory cytokine production in both cytotoxic and suppressor T cells.

### Enhanced Cytotoxicity, Impaired iCa^2+^ Influx and Insensitive iCa^2+^ Channels in Chronically Activated Tc1 From IL-10 KO Mice

**Figure 7:**
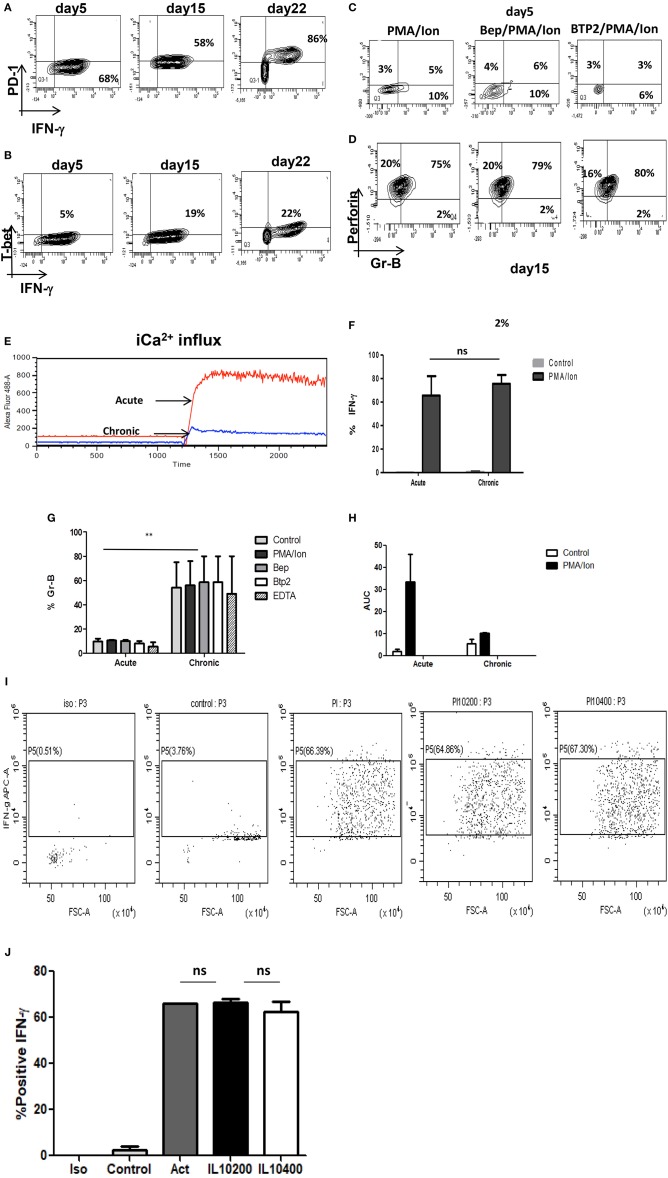
(i) Retention of cytotoxicity in IL-10 KO mice during chronic activation. Tc1 (Day 5), Tc15 (Day 15), Tc22 (Day 22) cells from IL-10 KO mice were reactivated with PMA/Ion and examined for IFN-γ, PD-1, and T-bet. Representative figures for PD-1 vs. IFN-γ **(A)** T-bet vs. IFN-γ **(B)** are shown. Tc1 (Day 5) and Tc15 (Day 15) cells were also examined for cytotoxic proteins Gr B vs. Perforin in the presence or absence of calcium channel inhibitors. Representative figure for Gr B vs. Perforin in PMA/Ion vs. Bep/PMA/Ion vs. BTP2/PMA/Ion are shown **(C,D)**. Tc1 and Tc15 cells were stained with Fluo-4 AM and kinetics of iCa^2+^ influx was recorded for about 30 min **(E)** post TCR stimulation and representative figure showing iCa^2+^ influx in Tc1 vs.Tc15 is shown. Summary of frequency of Tc1 and Tc15 cells producing IFN-γ **(F)** and Gr-B **(G)** in the presence or absence of calcium channel inhibitors are shown. Bar represents % positive cells and cumulative AUC data **(H)** representing iCa^2+^ influx from IL-10 KO mice is shown. (ii) IFN-γ secretion is not effected by exogenous IL-10 in IL-10 KO Tc15 cells- Tc15 (Day 15) from IL-10 KO mice were reactivated with PMA/Ion and examined for IFN-γ secretion upon addition of exogenous recombinant IL-10 at 200 ng/ml and 400 ng/ml concentration. Representative figure showing **(I)** increase in IFN-γ following activation with PMA/Ion to 66.3 from 3.76% in control. However, there is no significant change in IFN-γ production with addition of exogenous IL-10 at 200 or 400 ng/ml concentration. **(J)** Derived data showing insignificant change in IL-10 production following exogenous IL-10 addition. *p* < 0.5 (*p < 0.05, **p < 0.01, ***p < 0.001) considered as significant.

Tc1 cells execute their cytotoxic function by means of cytotoxic molecules that include amongst others Gr B and Perforin-C whose functions are reported to be impaired in chronic infections (5). As the combined function of IFN-γ, Gr B and Perforin-C is responsible for functional execution of cytotoxicity in Tc1 cells, we examined for Gr B expression in chronic activation, in IL-10 KO mice and with iCa^2+^ influx inhibitors. Our *ex vivo* acute activation demonstrated a direct correlation with IFN-γ and complete reverse relationship with IL-10 during chronic activation (Figures 7A–M). In the absence of IL-10 as the negative feedback regulator, IFN-γ and Gr B production was significantly elevated and most importantly their expression was not effected by iCa^2+^ influx inhibitors (Figures 7G–M). We found that IFN-γ secretion increased from 68 (acute) to 82% (chronic) and even after 22 days of activation the IFN-γ production was sustained at 86%. Expression of Gr B and Perforin-C were also highly significant as it increased from 14 (acute) to 75% in chronic activation. Most significantly we report that the iCa^2+^ influx was significantly lower in chronic activation of Tc1 cells as compared to their acute counterpart in IL-10 KO mice (Figure 7E). Interestingly PD-1 and T-bet expressions were also up regulated in IL-10 KO mice confirming loss of the negative feedback loop in their regulation (Figures 7A,B) and also confirming the seminal and singular role for IL-10 in down regulating immune responses. The up regulation of PD-1 in both wild type and IL-10 KO mice suggested that PD-1 signaling alone becomes redundant in the absence of IL-10. Further these results most crucially establish that proximal iCa^2+^ influx following TCR activation and downstream IL-10 production exquisitely and exclusively down-regulate(s) the pro-inflammatory status of the cytotoxic T cell. Proximal iCa^2+^ influx inhibition had no inhibitory effects on IFN-γ, Gr B or perforin-C in the absence of IL-10 as evidenced in the IL-10 KO experiment suggesting that IL-10 alone was the distal effector of immune suppression and regulation. Thus, our results show that iCa^2+^-IL-10 axis plays a critical role in modulating and fine-tuning cytotoxic immune responses during activation and chronic infections. Importantly downstream IL-10 alone determines loss of cytotoxicity and immune suppression and the fine-tuning by IL-10 is mandatorily determined from very early antigen presentation.

### Exogenous IL-10 Could Not Restore IFN-γ Production in IL-10KO Tc15 Cells

Our IL-10 KO data suggested that complete absence of IL-10 lead to higher IFN-γ production even in Tc15 cells and strongly suggested the central role of IL-10 in down-regulating immune response. Therefore, to further confirm and establish the role of IL-10 as the most important immune response down-regulator, we used exogenous recombinant IL-10 at 200 ng/ml and 400 ng/ml concentration (44). Surprisingly our result showed minimal and insignificant reduction in IFN-γ (Figures 7I,J) and suggests that exogenous IL-10 alone in the absence of an antigen mediated regulatory pathway was insufficient. These results validated our hypothesis that TCR signaling mediated immune responses have extreme fine tuning and inflammatory and anti inflammatory responses are determined and established very early.

### i[Ca^2+^] Flux, ROS, STAT's, IL-10, and CD8^+^ T Cell Plasticity

**Figure 8:**
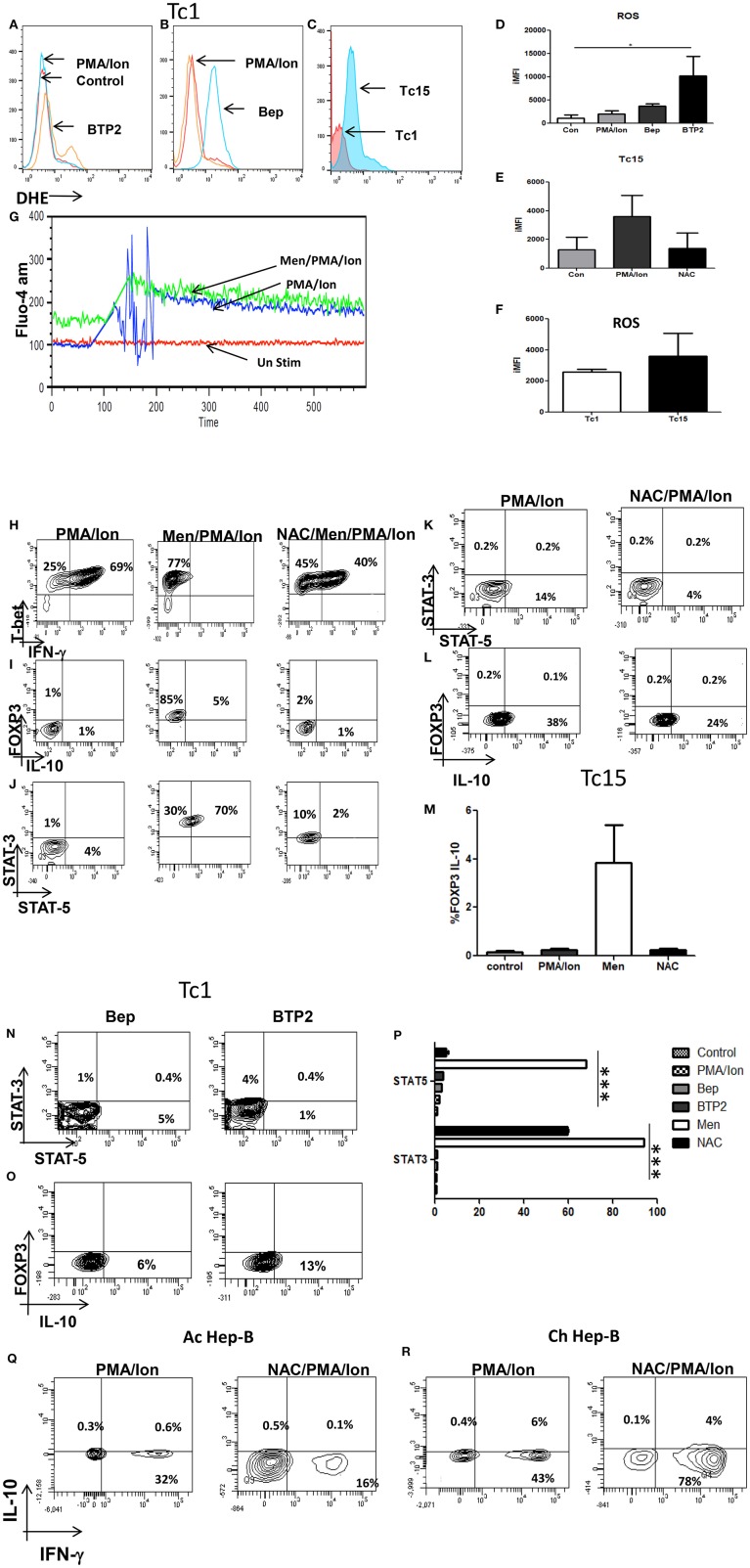
Reduction of iCa^2+^ flux in acute (Tc1) and chronic (Tc15) activation increases ROS production. Differentiated Tc1 cells were pre-loaded with DHE (5 um) for 30 min in medium without serum. Cells were then washed with PBS and treated with indicated concentration of Menadione for 15 min with or without NAC (5 Mm). In some cases, cells were treated with Menadione at indicated concentrations for 15 min and then DHE (5 uM) was added to the culture and incubated for 15 min. After incubation, cells were washed, re-suspended in PBS and oxidized DHE was detected by flow cytometry. Inhibition of iCa^2+^ flux through Bep and BTP2 increases ROS production in Tc1 cells **(A,B,D)**. Tc15 cells produce higher ROS when compared to their acute counterparts and was reduced following NAC treatment **(C,E,F)**. Treatment of Menadione had no effect on calcium influx **(G)**. (ii) Differentiated Tc1 cells were treated with 2.5 um Menadione with or without 5 mM NAC. After incubation cells were stained for T-bet-IFN-γ, FOXP3-IL-10 and STAT3-STAT5 molecules. Tc15 cells are treated with 5 mM NAC **(H)**. Menadione treatment reduced IFN-γ production significantly that is rescued upon NAC treatment **(I,J,M)**. FOXP3-IL-10 and STAT3-STAT5 expression increased upon menadione treatment and was lowered significantly upon treatment with NAC. Tc15 cells express significantly higher STAT3, STAT5 and IL-10 as compared to Tc1 cells **(K,L,P)**. Increase in STAT3, STAT5 and IL-10 expression following treatment with calcium channel inhibitors Bep and BTP2 **(N,O)**. IFN-γ production was reduced following treatment with anti-oxidant NAC in acute Hep-B whereas in chronic Hep-B it increases IFN-γ and reduces IL-10 production **(Q,R)**. All samples were tested for significance by using two-way ANOVA and *p* < 0.5 (*p < 0.05, **p < 0.01, ***p < 0.001) considered as significant.

After establishing the regulatory role of iCa^2+^ influx we then examined the role of Reactive Oxygen Species (ROS) and anti-oxidants in regulating phenotype change in terminally differentiated cytotoxic T cells and CD8^+^ T cells in chronic viral infections. Our results revealed that induction of ROS did not affect iCa^2+^ flux (Figure 8G) whereas reduction of iCa^2+^ flux through BTP2 and Bep increased ROS concentration (Figures 8A,B,D) indicating that iCa^2+^ flux worked upstream of ROS. Most importantly comparison of ROS concentration between acute and chronically activated CD8^+^ T cells reveals that chronic activation produced higher amounts of ROS as compared to their acute counterpart that is brought down by treatment of antioxidant NAC (Figures 8C,E,F). We further investigated how reduced i[Ca^2+^] flux influenced the JAK-STAT pathway and our results revealed that CRAC channel inhibition by BTP2 increased STAT-3 expression from 1 to 4% while Bep increased STAT-5 expression from 0.5 to 5% (Figures 8N–P) indicating involvement of ROS and calcium in regulating IL-10 producing anti-inflammatory pathway through STAT-3/STAT-5 axis. Our data suggested that both reduced iCa^2+^ flux and increased ROS leads to FOXP3/IL10 positivity. Treatment with Menadione, a ROS inducer, reduced the T-bet/IFN-γ double positive population significantly while NAC treatment partially reversed the trend, establishing certain plasticity in cytotoxic T cells through ROS (Figure 8H). Interestingly on the other hand, Menadione treatment increased FOXP3 expression significantly that was completely restored with NAC (Figures 8I,J). Comparison of these results with chronically activated CD8^+^ T cells revealed dual increase in STAT3/STAT5 although STAT5 expression was much more affected. Interestingly treatment of chronically activated CD8^+^ T cells with ROS scavenger reduced the IL-10 production from 38 to 24% (Figure 8L). These results indicated a differential role for STAT-3 and STAT-5 in stabilizing the T suppressor phenotype and most crucially that ROS and iCa^2+^ flux could alter CD8^+^ T cell phenotypes.

Further for proof of principle we examined CD8^+^ T cells from acute and chronic Hep-B patients. Our data suggested that treatment of CD8^+^ T cells with NAC from acute Hep-B/C patients lead to reduced IFN-γ production (Figures 8Q,R) while NAC treatment in chronic activation lead to increase in IFN-γ production from 43 to 78% while IL-10 production was reduced from 6 to 4%. The reduction in IFN-γ production in acute Hep-B/C infection indicated that a window of cellular ROS is required for IFN-γ production below and above which its expression is significantly altered. On the other hand elevated ROS levels are required for IL-10 production. Taken together our data suggests that reduced iCa^2+^ flux lead to increased ROS production that in turn express IL-10 through STAT3/STAT5 axis leading to impaired immune response during chronic infections.

## Discussion

Chronic intra cellular infections are caused by unproductive immune responses to viruses and this continuous inefficient activation leads to T cell exhaustion (10, 20, 45, 46) whereby they lose their ability to secrete pro-inflammatory cytokines and cytolytic proteins (47, 48). Tc1 cells known to be important for clearance of viral infections through secretion of IFN-γ and cytolytic molecules Gr B and Perforin-c (2, 49, 50) have been shown to change phenotype and secrete IL-10 and this compromised cytotoxic status has been shown to precipitate chronic viral infection(s). Blockade of IL-10R (6) and ablation of Treg cells (11) have been known to rescue exhausted Tc1 cells during chronic viral infections indicating the suppressive role of IL-10 in the impairment of immune responses (8, 10, 14, 20, 51, 52). Interestingly these exhausted T cells have been extensively characterized but the proximal signaling pathway post TCR activation by which the exhausted Tc1 cells convert (10, 53–56) is still undefined. Our results show a direct link between altered iCa^2+^ influx (57), ROS, NFAT1 translocation (30, 58) and the flipping of pro and anti-inflammatory cytokines and further provide strong evidence that iCa^2+^ influx (29, 59)-ROS-IL-10 nexus dictates the conversion of effector Tc1 cells into T suppressive subsets through STAT3/STAT5 axis.

Chronic activation of cytotoxic T cells subverts their cytotoxic capabilities to an immune-suppressive type and our results are in accordance with previous studies (2, 49). The progressive and gradual flip of a pro-inflammatory cytotoxic cell type generated during acute activation to an anti-inflammatory immune suppressor cell type encountered in chronic activation was seen in both our murine and human CD8^+^ T cells (11, 17). This flip from a cytotoxic to a suppressor phenotype is evidenced even at the transcriptional level as cytotoxic signature transcription factor T-bet expression apart from IFN-γ and Gr B are markedly reduced (22). As the signaling cascade downstream of TCR activation required for the above two functionally different cytokines are quite different we hypothesized that chronic activation would necessitate a complete change of TCR downstream signaling events in Tc1 cells during their conversion to a T suppressor type. iCa^2+^ influx along with ROS mark two of the earliest signaling events downstream of TCR activation (59–62) and it has been reported that the dynamics of iCa^2+^ and ROS in T cells lead to differential cytokine production (63). Our data presented here suggests that iCa^2+^ flux is reduced and ROS is increased significantly during chronic activation in both murine and in persistent human infection and clearly co relate with an immune suppressive profile. Change in both proximal iCa^2+^ influx and ROS by use of iCa^2+^ inhibitors, oxidant/anti –oxidants in acute activation and chronic activation in mice CD8^+^ T cells or persistent infections in humans clearly down regulates pro-inflammatory cytokine IFN-γ and up regulates IL-10 production. Crucially the comprehensive loss of sensitivity to iCa^2+^ influx inhibition in CD8^+^ T cells from IL-10 KO mice implied a mandatory requirement for IL-10's presence in the course of an immune response. In addition the completely unaffected nuclear translocation of NFAT1 in chronically activated CD8^+^ T cells from IL-10 KO mice very strongly suggested a proximal iCa^2+^ influx and distal IL-10 axis in down regulating cytotoxicity. The loss of IL-10 in the genetically altered mice we used clearly demonstrated the connection between iCa^2+^ influx, distal IL-10, NFAT1 translocation and the subsequent cytotoxicity. The dysregulation in the chronic activation leading to exacerbated cytotoxicity points to the mandatory requirement of IL-10 to fine-tune the immune response during an infection. Additionally the inherent capability to convert a cytotoxic T cell to a suppressor phenotype through iCa^2+^ influx inhibition marks a pivotal step in down regulating uncontrolled or accidental cytotoxic response and may even be operational physiologically. These seminal points and the crucial finding that chronic activation or infections can completely inhibit CD8^+^ T cell iCa^2+^ influx mark it as an early diagnostic marker to distinguish cytotoxic and immune suppressive status of CD8^+^ T cells. The proof of principle established in both mouse and human studies give significant credence to our findings.

Most notably in our inhibitor studies where iCa^2+^ influx was reduced by non-selective plasma membrane calcium channel inhibitor Bepridil hydrochloride and CRAC channel inhibitor BTP2, there was a direct co relation with reduced IFN-γ and Gr B and significantly increased IL-10 production. Contrastingly with inhibition of intracellular calcium channel Ryanodine receptor and SERCA pump through Amlodipine Besylate and Thapsigargin; neither the iCa^2+^ nor was IFN-γ production affected. Further with no significant IL-10 production it suggested that iCa^2+^ through plasma membrane and CRAC channels were the most critical for TCR downstream signaling events leading to inhibition of pro inflammatory and production of anti-inflammatory cytokine(s). Crucially when iCa^2+^ influx was not affected IFN-γ and Gr B production were unaffected implying that TCR mediated early iCa^2+^ influx through plasma membrane and CRAC channel determined cytotoxicity. Our results with CD8^+^ T cell activation profiles, iCa^2+^ inhibitors, IL-10 KO mice and human samples clearly demonstrate an implicit and direct co relation between iCa^2+^ influx and cytotoxicity. Inhibition of iCa^2+^ influx explicitly was associated with chronic activation and resulted in gain of immune suppressive phenotype except when IL-10 was absent, suggesting IL-10 as the distal mediator to immune suppression.

Further induction of ROS through Menadione in Tc1 subtype led to significant increase in FOXP3/IL-10 and reduced T-bet/ IFN-γ production and this reciprocal cytokine change was accompanied by altered STAT3/STAT5 expression. Intriguingly treatment with the anti-oxidant NAC restored T-bet population while reduced the STAT3/STAT5 population but not restore IFN-γ production indicating the involvement of other signaling molecules. Interestingly inhibition of iCa^2+^ flux also displayed higher ROS and STAT3/STAT5 expression substantiating the relationship between iCa^2+^ flux, ROS, STAT molecules. STAT3 is known to be responsible for IL-10 production and IL-10 is the key effector molecule through which T suppressors execute their function through STAT5 that is required to boost the immune-suppression function of Treg cells (64). Not surprisingly chronic activation of Tc1 cells also displayed higher ROS, STAT3/STAT5 further substantiating our hypothesis that increase in ROS and decrease in iCa^2+^ flux led to a T suppressor subtype and iCa^2+^ flux acts upstream of ROS as induction of ROS didn't effect iCa^2+^ flux whereas reduced iCa^2+^ flux increased ROS.

The most significant and latent finding in our study is the understated seminal role for IL-10 in immune regulation and suppression. The abnormally high expression of T-bet, IFN-γ, Gr B, and Perforin-c in IL-10 KO mice over wild type mice clearly suggested the critical role played by IL-10 in immune regulation and in fine tuning immune responses. Even significantly increased PD-1 and LAG-3 protein were completely incompetent in immune suppression (9, 10, 15, 65) in these cells suggesting the cardinal and singular role for IL-10 in immune regulation. The absence of IL-10 in these studies further compounds earlier interpretation of the supposed roles of PD-1 and LAG-3 proteins considering that earlier human studies have also shown that PD-1 need not necessarily mark exhaustion (66). However, completely inhibited iCa^2+^ influx profile in the chronically activated CD8^+^ T cells and their complete insensitivity to iCa^2+^ influx inhibitors in the IL-10 KO derived ones suggested that iCa^2+^ influx was fine-tuned by IL-10 during an immune response and its loss may lead to a severe and uncontrolled cytotoxic inflammation and subsequent immune-pathology.

Here the suppressive function of IL-10, secreted as a result of conversion of effector T cell into T sup cell following chronic TCR stimulation is not antigen specific contrary to those produced by co-culture of T cells with natural CD4^+^CD25^+^ T cells (67, 68). In both the cases, the T sup cells execute their function through IL-10 and are contact independent. The general suppressive activity of T sup cells generated in our study is significant in case of chronic infections as the phenomena leads to significant reduction of effector T cells required for clearing the infection and importantly. Moreover, these converted T sup cells can also affect the general immune homeostasis as IL-10 inhibits strong Tc1 and Th1 response and additionally IFN-γ producing effector T cells are primarily inhibited and then converted into IL-10 producing T sup cells.

Amongst the Transcription factors T-bet and FOXP3 that we studied we report that T-bet, the signature transcription factor of Tc1 type was reduced significantly during chronic activation while FOXP3 a signature transcription factor for CD4^+^ Treg type (69) was not majorly altered during chronic activation or with the use of inhibitors and did not seem to reflect immune suppressive functions or capabilities of the T suppressor. Additionally though T-bet expression was reduced during chronic activation, iCa^2+^ influx inhibitors did not reduce their expression significantly during acute activation. So we concluded that the reduction in IFN-γ with iCa^2+^ influx inhibitors could be T-bet independent during acute activation and that IL-10 production from these cells could differ from pathways elicited by the CD4^+^ Treg type. We further hypothesize that the time period for calcium channel inhibition during acute activation may not be sufficient enough to affect Treg transcriptionally nor was it required to down regulate it to affect IFN-γ and Gr B levels.

The single most relevance of this study and proof of principle are demonstrated in acute and chronic infection in human Hepatitis B where we found that iCa^2+^ influx in CD8^+^ T cells was completely abrogated with a significant increase in IL-10 and decrease in IFN-γ. Amongst T cell compartments we examined in chronic Hep B patients, Memory and Effector T cells demonstrated a clear IL-10 bias as compared to their acute counterparts and control. Importantly regression analysis showed that IL-10 negatively regulates IFN-γ production in chronic Hep-B and most significantly treatment with the antioxidant NAC restored IFN-γ production while reducing IL-10 levels. The role of IL-10 in the pathogenesis of Hepatitis-B is paradoxical where some authors report that effector CD8^+^ T cell-derived IL-10 enhances acute liver immunopathology whereas some others report; IL-10 mediated liver immunopathology during chronic Hep-B infection in Indian population (44, 70). As in *M tb* infections a strong Tc1 response is mandatory for viral clearance with collateral damage while IL-10 minimizes damage and allows the pathogen to grow *albeit* slowly. In Hep B however since viral replication is high, IL-10 secreting cells do not allow viral clearance subsequently leading to liver pathology while a robust Tc1 response would allow viral clearance as is mandated for any viral infection clearance.

In summary our study demonstrates that reduced iCa^2+^ influx lead to production of the suppressive cytokine IL-10 through which IFN-γ and Gr B expression are down regulated. Further our study in human Hep B samples in good co-relation with murine data suggests that iCa^2+^ influx could be used as a potential diagnostic marker for cytotoxic or suppressor T cell.

## Data Availability Statement

The human clinical data is stored at SUM, Hospital and with the authors and on request for this, ethical, legal, and privacy consent will be requested from both the doctor and patient to share data. Our mouse data can be made available to any researcher on request.

## Ethics Statement

The studies involving animals were carried out in accordance with the recommendations of the Committee for the Purpose of Control and Supervision of Experiments on Animals (CPCSEA), and the ILS animal ethics committee. The mice were taken from ILS animal house as per the institute guidelines.

## Author Contributions

SM designed, carried out and analyzed the experiments, and prepared the manuscript. PB prepared and handled human samples. ND performed clinical diagnosis and handled human samples. PN prepared KO mice confirmation and designed KO experiment inputs. SD designed and analyzed the experiments, prepared the manuscript and provided overall supervision.

### Conflict of Interest

The authors declare that the research was conducted in the absence of any commercial or financial relationships that could be construed as a potential conflict of interest.

## Abbreviations

Bep: Bepridil Hydrochloride
Amlo: Amlodipine Besylate
Thap: Thapsigargin
Ac: Acute
Ch: Chronic.
