# Supplementary material for: iCa2+ Flux, ROS and IL-10 Determines Cytotoxic, and Suppressor T Cell Functions in Chronic Human Viral Infections
Source: Front Immunol. 2020 Mar 6;11:83. doi: 10.3389/fimmu.2020.00083 (PMC7068714; doi:10.3389/fimmu.2020.00083)
Supplement: Supplementary file 1 [file Data_Sheet_1.pdf]

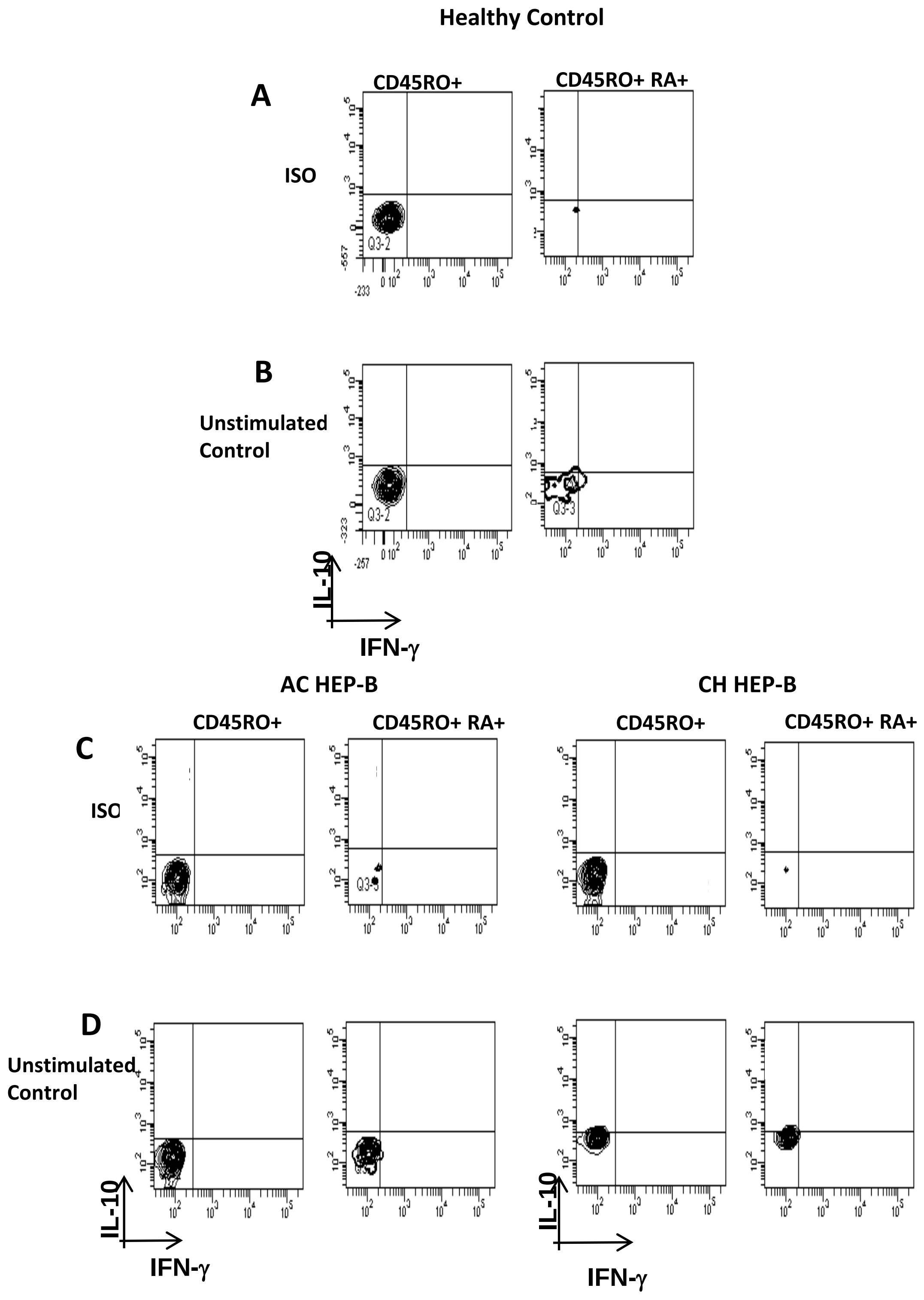

Mohanty et al., sup Fig. 1- Acute and Chronic Hep B patients display varied IFN-γ and IL-10 secretion patterns. (A) Representative figures showing isotype for CD45 RO+ and CD45RORA+ and (B) the corresponding unstimulated controls for CD8+ T cells from healthy controls. (C) Representative figure showing isotype for CD45 RO+ and CD45RORA+ and (D) the corresponding unstimulated controls for CD8+ T cells from acute and chronic Hep-B patients.

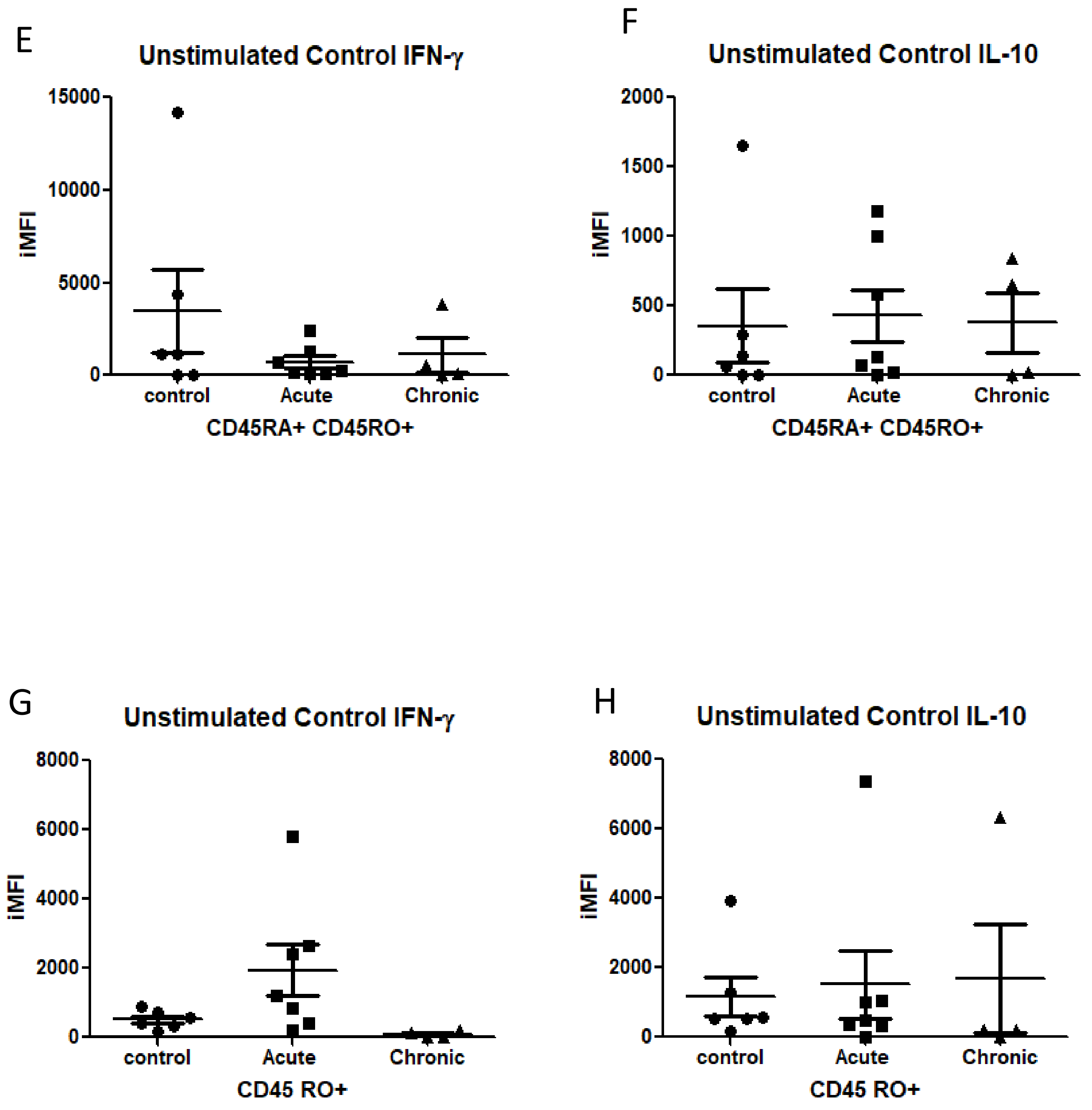

Mohanty et al., sup Fig. 1- Acute and Chronic Hep B patients display varied IFN-γ and IL-10 secretion patterns. (E,F) Cumulative data representing comparison of IFN-γ and IL-10 production by unstimulated controls of effector CD8+ T cells from healthy, acute and chronic Hep-B patients. (G,H) Cumulative data representing comparison of IFN-γ and IL-10 production by unstimulated controls of memory CD8+ T cells from healthy, acute and chronic Hep-B patients

Isotype

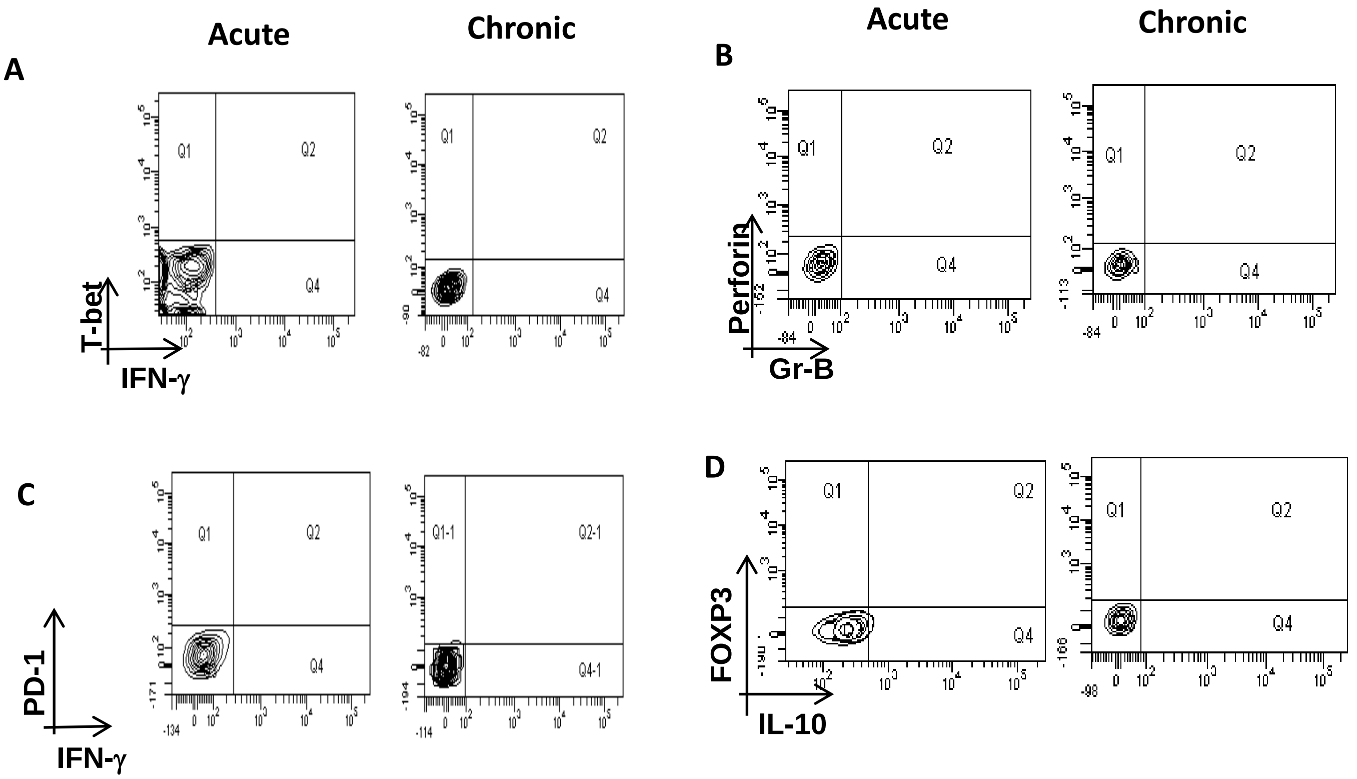

Mohanty et al., sup Fig. 2- Chronic activation of Tc1 leads to impaired cytotoxic function and increased IL-10 production. (A,-D) The isotype controls for T-bet/IFN-γ, Perforin/Gr-B, PD-1/IFN-γ and FOXP3/IL-10 for acute and chronic activated murine CD8+ T cells respectively.

Isotypes for IL-10 KO experiment

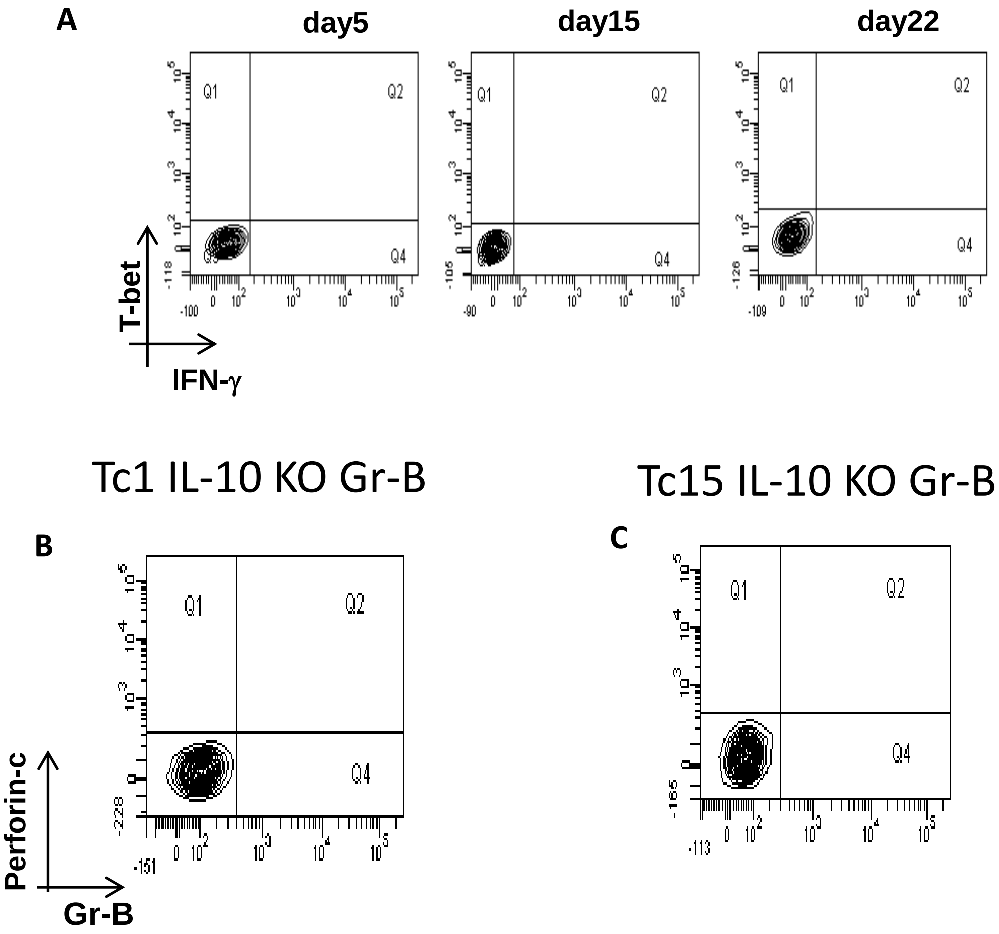

Mohanty et al., sup Fig. 3- IL-10 KO mice retain cytotoxic function in chronically activated CD8$^+$ T cells. (A) Isotype controls for T-bet/IFN-γ for day 5, day15 and day22 activation of CD8+ T cells from IL-10KO mice. (B,C) Representative figure showing isotype control for perforin-c/Gr-B in acute (Tc5) and chronic(Tc15) activation of CD8+ T cells from IL-10 KO mice.

Gating strategy for STATS

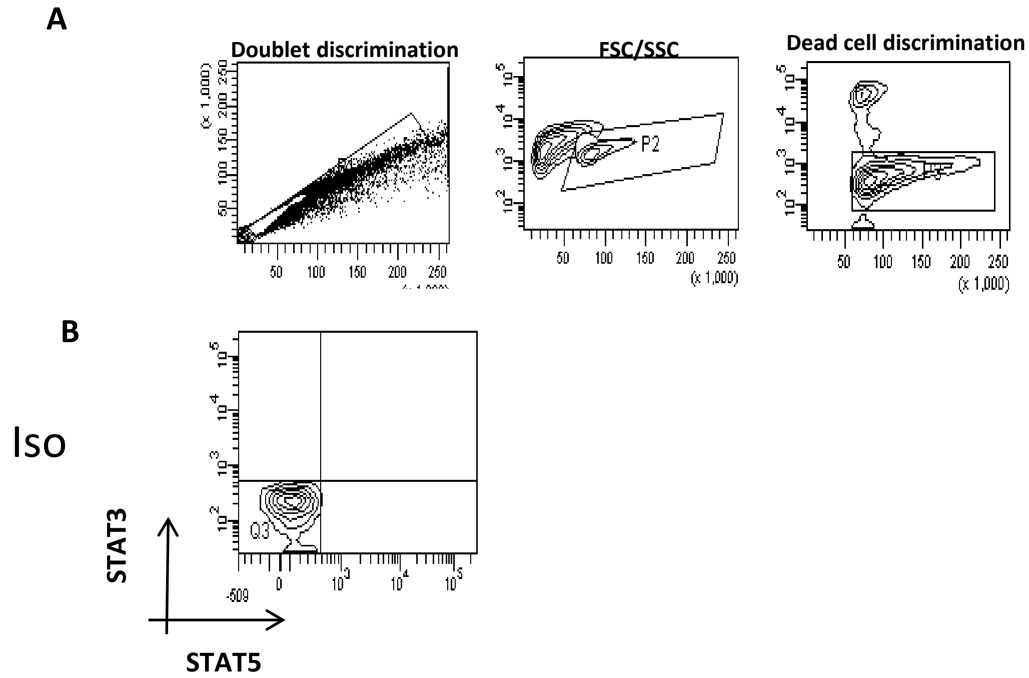

Mohanty et al., sup Fig. 4- Induction of ROS converts Tc1 subtype into T-sup phenotype through STAT3-STAT5 axis. (A) Schematic representation of gating strategy for analysis of STAT molecules. (B) Representative figure showing isotype control for STAT3/STAT5 molecule for Tc1 cells.

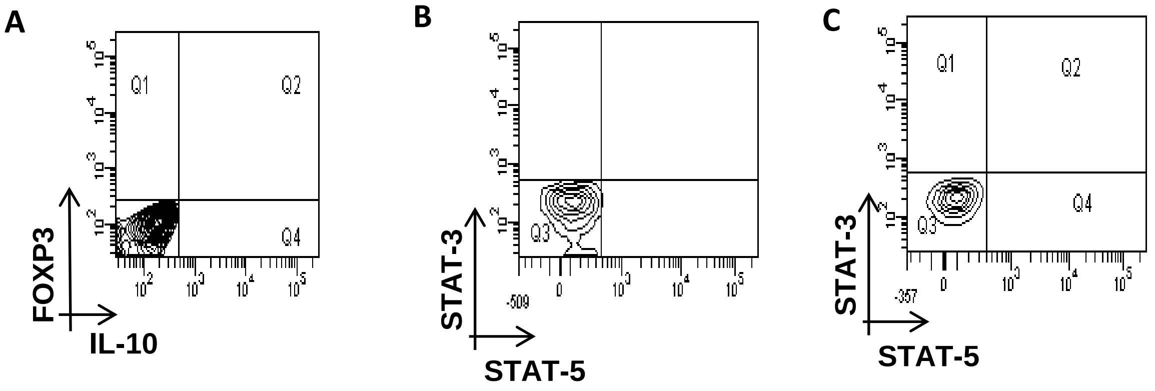

Isotype for Fig 8I Isotype for Fig 8J Isotype for Fig 8K

Mohanty et al., sup Fig. 4- Induction of ROS converts Tc1 subtype into T-sup phenotype through STAT3-STAT5 axis. (A-C) Representative figure showing isotype controls for FOXP3/IL-10 in Tc1 cells and for STAT3/STAT5 of Tc1 and Tc15 murine CD8+ T cells respectively.

Isotypes for Bep and BTP2 in Tc15 cells

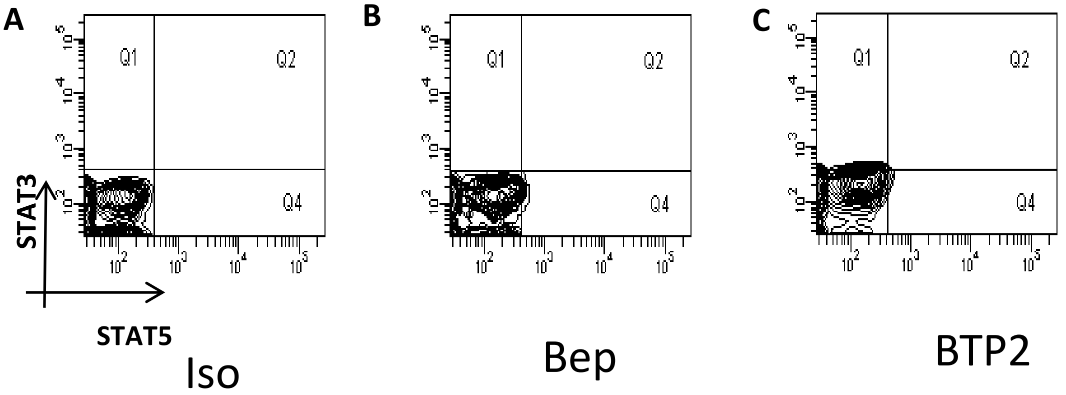

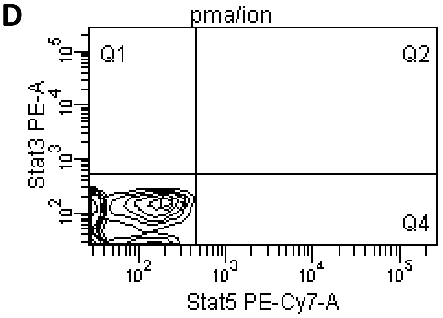

Mohanty et al., sup Fig5- Induction of ROS converts Tc1 subtype into T-sup phenotype through STAT3-STAT5 axis. (A-C) Representative figure showing isotype control and unstimulated control for Bep and BTP2 respectively. (D) Representative figure showing the activation control (PMA/Ion) for induction of STAT3/STAT5 following menadione treatment in Tc1 cells.
